# Supplementary material for: Subtherapeutic Doses of Vancomycin Synergize with Bacteriophages for Treatment of Experimental Methicillin-Resistant Staphylococcus aureus Infective Endocarditis
Source: Viruses. 2022 Aug 16;14(8):1792. doi: 10.3390/v14081792 (PMC9412893; doi:10.3390/v14081792)

SUPPLEMENTAL MATERIAL

SUPPLEMENTAL MATERIAL AND METHODS

Bacterial strains, growth conditions, and antibiotic susceptibility testing.

S. aureus AW7 was stored in TSB (BD DifcoTM, Becton Dickinson, Sparks, MD) containing 20% (v/v) glycerol at -80°C and sub-cultured on TSA plates to ensure purity before testing. For liquid cultures, TSB was inoculated with at least five isolated colonies and incubated for 24 h with agitation (200 rpm) at 37°C.

Vancomycin was purchased from Merck KGaA, Darmstadt, Germany. The MICs of vancomycin were determined in Muller Hilton Broth (MHB, Becton Dickinson, Sparks, MD) using a standard micro-dilution procedure [25].

Bacteriophages.

The *Podoviridae* phage 66 and *Herelleviridae* phage vB_SauH_2002 genomes are publicly available (Genbank accession no. NC_007046 and MW528836, respectively). To produce large quantities of phages, amplification was performed using MSSA Laus102 as propagation strain as previously described [14]. The equimolar phage cocktail at 10 $\log_{10}$ PFU/mL was assembled after adjusting the concentration of each phage to 10 $\log_{10}$ PFU/mL.

In vitro turbidity assays.

Bacteria were used in exponential growth phase. Ten mL of TSB were freshly inoculated with 100 µL of an overnight culture of MRSA AW7 and placed in an incubator at 37°C under 200 rpm until OD_{600nm} reached 0.6 (ca. 8 $\log_{10}$ CFU/mL). Ten µL of this bacterial suspension (ca 6 $\log_{10}$ CFU) were added to wells of 96-well microtiter plates (Thermo Scientific, USA) already filled with 290 µL of TSB and various concentrations of either phages alone or phage cocktail (final MOI of 0.01, 0.1, 1, 10, and 100). The microtiter plates were incubated at 37°C in an Elx808IU absorbance microplate reader (BioTek®, Sursee, Switzerland) and the OD_{600nm} was measured every 10 minutes for 24 h. Before each measurement, the microtiter plates were shaken for three seconds to homogenize the solution. All experiments were performed in triplicate.

Time-kill curve assays.

One hundred µL of an overnight culture of MRSA AW7 was used to inoculate 10 mL fresh TSB. This solution was placed in an incubator at 37°C under 200 rpm shaking until the OD_{600nm} reached 0.6. The culture was then diluted 1:100 in 10 mL fresh TSB containing (i) 6 $\log_{10}$ PFU/mL of phage vB_SauH_2002, (ii) 6 $\log_{10}$ PFU/mL of phage 66, (iii) an equimolar concentration of phage vB_SauH_2002 and phage 66 to reach 6 $\log_{10}$ PFU/mL, (iv) 2x MIC of vancomycin (2 µg/mL), (v) a combination of both antibiotic and phage treatments. The tubes were incubated at 37°C under

200rpm. The concentration of bacteria was determined at 0 h, 2 h, 4 h, and 24 h after inoculation (limit of detection 2 log_{10} CFU/mL). To avoid carry over of phages, samples were diluted in 1x PBS (pH 3) before plating on TSA plates. All experiments were performed in triplicate.

Additional information related to the EE model.

Randomization. Randomization of animals in groups was done using the online tool Research Randomizer (https://randomizer.org/)

Outcomes. The primary outcome was bacterial load in cardiac vegetations 24 h and 48 h after the onset of treatments. Secondary outcomes were phage loads 24 h and 48 h after the onset of treatments in cardiac vegetations, blood, spleen, liver, and kidneys. An additional outcome was the presence of phage-resistant clones in the cardiac vegetations of rats given the phage cocktail/vancomycin combination treatment for 48 h. Outcome assessment methods are described in the Supplemental Material.

Vancomycin dosing regimen. For up to 48 h, rats received a sub-therapeutic IV dose of vancomycin mimicking human kinetic treatment (0.5 g b.i.d instead of 1 g b.i.d, which would have been the recommended daily dose [16]). The administration protocol consisted in the infusion of a 1.25 mg/mL solution of vancomycin diluted in saline. The pump cycle for 24 h delivery of the treatment was as follows: 2.3 mL/h for 30 minutes followed by 1.3 mL/h for 1 hour 30 min; 0.54 mL/h for 1 h; 0.28 mL/h for 3 h; and 0.18 mL/h for 6 h [28]. After this cycle, a new infusion cycle was started.

Phage cocktail dosing regimen. As previously described [14], the equimolar two-phage cocktail consisted of phage vB_SauH_2002 + phage 66 at 10 log_{10} PFU/mL each. The regimen consisted of a 1 mL bolus followed by continuous infusion at 0.3 mL/h for 24 hours (each rat receiving a total of 10.91 log_{10} PFU) or as a 1 mL bolus followed by continuous infusion at 0.3 mL/h for 48 hours (each rat receiving a total of 11.19 log_{10} PFU).

Criteria for euthanasia. Animal welfare was assessed at least twice a day with an in-house welfare score sheet for rodents (see below). Animals were excluded from randomization if we suspected that the catheter placed into the heart through the carotid artery was not properly inserted. Animals were euthanized humanely according to the score and status of the animal as indicated below (termination criteria). The mortality rate after surgery was 10%, and six rats were excluded before infection. Moreover, eight rats were further excluded at the end of the experiment because the catheter was not properly inserted.

Welfare score sheet used in the *in vivo* experiment of EE rats.

	Score			
	0	1	2	3
Haircoat	Normal Well groomed	Fur ruffling	General lack of grooming	Hunched up with matted fur
Posture	Normal	Sporadic hunchback posture	Frequent hunchback posture	Head on cage floor
Activity	Normal	Decreased activity after slight stimulation	Significant decreased activity after moderate stimulation	Lethargy after moderate stimulation
Breath	Normal	Shallow	Labored breathing	Breathing noises
Behavior	Normal	Isolated from cage mates*		Convulsion

Termination criteria:
Score of 0: no action.
Score of 1: animal is observed twice daily. If animal does not return to normal within 48 hours it will be euthanized.
Score of 2: animal is observed three times daily. If animal does not return to normal within 12 hours it will be euthanized.
When an animal reaches a score of 3, either cumulative or in one observable criteria, it will be immediately euthanized.
*this score is not applicable for animals that are isolated in a cage, for instance animals equipped with a “swivel” system.

Blinding procedure. The rats receiving saline and phages, or saline and antibiotics were connected to the same pumps, rendering the masking of group/treatment assignment challenging. Moreover, blinding at this step was unnecessary since performed during assessment of microbiological outcome.

Bacterial loads in cardiac vegetations. The presence of macroscopic cardiac valve vegetations was visually validated before being dissected from the heart. After being weight, vegetations were further mechanically homogenized in 1 mL saline. The homogenates were serially diluted and plated in triplicate on TSA plates for bacterial counting. Colonies were counted after an overnight incubation at 37°C. Remaining vegetation homogenates were stored at -80°C after the addition of 20% (v/v) glycerol. Potential phage or vancomycin carry over was avoided through serial dilutions.

Outcomes. The primary outcome was the bacterial loads in cardiac vegetations 24 h or 48 h after the onset of treatments. Secondary outcomes were phage loads 24 h or 48 h after onset of treatments in cardiac vegetations, blood, spleen, liver, and kidneys. An additional outcome was the presence of phage-resistant clones in the cardiac vegetations of rats that received the phage cocktail/vancomycin combination treatment for 48 h.

Phage loads in cardiac vegetations, organs, and blood. Whole blood samples (1 mL) were collected from the vena cava, supplemented with 100 IE of heparin (Liquemin®, Drossapharm AG/SA, Basel, Switzerland) and serially diluted in saline. After dissection, organs were mechanically homogenized in weight-adapted volumes of saline (1 mL for cardiac vegetations, 2 mL for spleen, liver, and kidney). Phage loads were determined using a classical double-layer assay. Plates were incubated at 37°C and PFU were manually counted the following day.

List of animals in groups.

**Number of animals**	**Onset of treatment**	**Saline 24 h**	**Phage cocktail 24 h**	**Vancomycin 24 h**	**Phage cocktail + vancomycin 24 h**	**Phage cocktail + vancomycin 48 h**
**Considered (N)**	8	8	8	10	8	10
**Dead after surgery**	0	1	1	1	2	1
**With not properly placed catheters**	2	1	1	1	2	1

Determination of phage-resistance patterns of *S. aureus* AW7 clones recovered *in vivo*.

The phage-resistance patterns of the clones recovered *in vivo* from the rat cardiac vegetations were determined with diluted drop test assays. Cardiac vegetation homogenates (100 µL) were plated on TSA and incubated overnight at 37°C. Two days later, single colonies were re-suspended in 5 mL fresh TSB and incubated overnight at 37°C. Overnight bacterial cultures were mixed 1:100 with 15 mL of TSB soft-agar and poured into Petri dishes. The bacterial lawns were then spotted with 5 µL of serial 10-fold dilutions of each phage suspension (vB_SauH_2002, phage 66, and the phage cocktail) and incubated at 37°C overnight. The results were scored the next day according to the observed lysis phenotypes. Absence and presence of lysis were considered definitive of a resistant phenotype (R) and a susceptible phenotype (S), respectively (Figure S1).

Bacterial genomic DNA extraction and purification.

The protocol used to extract and purify genomic DNA from *S. aureus* AW7 and the different mutants sequenced in this work was adapted from Bae et al. 2008 [23]. Briefly, 3 mL of an overnight culture of *S. aureus* were centrifuged at 13’000 rpm for 1 minute and the pellet resuspended in 50 µL TE buffer containing 1 mg/mL lysostaphin (Merck KGaA, Darmstadt, Germany). After 30 minutes of incubation at 37°C, 300 µL of Nuclei Lysis Solution (Promega, Madison, WI, USA) was added before incubation at 80°C for 30 min. After cooling the sample to room temperature, 2 µL of PureLink™ RNase A (ThermoFisher Scientific, Waltham, MA, USA) was added and the tube further incubated for 30 min at 37°C. 100 µL of Protein Precipitation Solution (Promega, Madison, WI, USA) was added and the mixture incubated on ice for 5 min before centrifugation at 13’000 rpm, 4°C for 10 min. The supernatant was transferred to a clean Eppendorf tube and 300 µL of room temperature isopropanol (ThermoFisher Scientific, Waltham, MA, USA) was added before gentle repeated manual inversion.

The Eppendorf tube was further centrifuged at 13’000 rpm, 4°C for 10 min. The supernatant was discarded and 750 µL of room temperature 70% ethanol (ThermoFisher Scientific, Waltham, MA, USA) was added to the pellet. After several manual inversions, the tube was centrifuged at 13’000 rpm, 4°C for 10 min. The supernatant was discarded and after complete drying at 37°C, the pellet was resuspended in 50 µL ultra-pure water. DNA concentration was measured using the Quantus™ fluorometer and QuantiFluor® dsDNA Dye System (Promega, Madison, WI, USA) following the manufacturer’s recommendations.

Bacterial genome sequencing, assembly, and analysis.

For each purified bacterial DNA, a genomic library was prepared with an optimized protocol and standard Illumina adapter sequences. Full genome sequencing was performed with Illumina technology, NovaSeq 6000 (read mode 2 x 150 base pairs). Both processes were performed at Eurofins Genomics Germany GmbH (Ebersberg, Germany). Reads were assembled and contigs annotated using the PATRIC pipeline for assembly and annotation, respectively (https://www.patricbrc.org/). Comparative genomic was performed with the PATRIC variation analysis tool set to default parameters.

SUPPLEMENTAL TABLES

Supplemental table S1. Phage-resistance patterns of clones recovered *in vivo* from the vegetations of MRSA AW7-infected rats treated for 48 h with the phage cocktail/vancomycin combination.

Rat N°	Log CFU/g vegetations	N° of clones that grew in TSB	Phage-resistance patterns	
			**SSS**	**SRS**
2	3,1	2	2	
3	< detection limit	n.a.		
5	2,6	0		
6	4	5	5	
13	4	0		
14	3,1	0		
16	< detection limit	n.a.		
17	9,1	13	11(4)	2(2)
18	2,8	2	2	
19	8,2	10	10	
20	9,3	21	17(2)	4(4)
		**Total**	**47**	**6**

n.a., not applicable.

The three capital letters describing the phage-resistance patterns (SSS or SRS) correspond to the susceptibility of the clone to phage vB_SauH_2002, phage 66 and the phage cocktail, respectively. S, Susceptible; R, Resistant.

The number in brackets represent the number of clones whose genomes were fully sequenced.

Supplemental table S2. Detailed lists of mutations identified in the genomes of representative clones recovered from the vegetations of rats treated for 48 h with the phage cocktail/vancomycin combination.

A. SSS clones

17C7

Contig	Pos	Score	Ref_nt	Var_nt	Ref_aa_pos_change	Frameshift	Gene N°	Function
1	403394	3921.99	acgaaaaaatct	aCGAAAAAAATct	Thr120_Lys121fs	yes	385	Transposase, IS4 family
1	403435	15751.2	gcc	gTc	Ala109Val		385	Transposase, IS4 family
1	403568	167.07	agt	Ggt	Ser65Gly		385	Transposase, IS4 family
3	125623	6677.62	gtc	gGc	Val41Gly		993	Transposase, IS4 family
7	80346	8635.98	tggatggtttac	tgGATGGATGGTttac	Trp2_Met3fs	yes	1570	Integral membrane protein
11	87939	14434.7	aagaaagta	AAGAAAAGta	Lys154_Lys155fs	yes	2040	Transposase, IS4 family
11	87948	19534.5	ttggtgcgg	ttTGTGTgg	LeuValArg151PheValTrp		2040	Transposase, IS4 family
11	87971	19095.0	agt	aAt	Ser145Asn		2040	Transposase, IS4 family
11	87989	11532.3	gataattcaatttttattgatggt	AATAATTCAATTTTTATTGATGGt	Asp132Asn		2040	Transposase, IS4 family
11	88044	9730.58	ttctat	ttCCat	Tyr121His		2040	Transposase, IS4 family
11	88121	14594.7	ctt	cCt	Leu95Pro		2040	Transposase, IS4 family
11	88131	6380.24	gcccaagat	gcTCAAAat	AlaGlnAsp90AlaGlnAsn		2040	Transposase, IS4 family
19	19253	13276.3						

17C8

Contig	Pos	Score	Ref_nt	Var_nt	Ref_aa_pos_change	Frameshift	Gene N°	Function
1	403435	5400.93	gcc	gTc	Ala109Val		385	Transposase, IS4 family
1	403568	406.613	agt	Ggt	Ser65Gly		385	Transposase, IS4 family
3	125686	876.1	ggt	gAt	Gly20Asp		993	Transposase, IS4 family
7	80346	9589.88	tggatggtttac	tgGATGGATGGTttac	Trp2_Met3fs	yes	1570	Integral membrane protein
11	87939	14165.3	aagaaagta	AAGAAAAGta	Lys154_Lys155fs	yes	2040	Transposase, IS4 family
11	87948	17743.6	ttggtgcgg	ttTGTGTgg	LeuValArg151PheValTrp		2040	Transposase, IS4 family
11	87971	18186.5	agt	aAt	Ser145Asn		2040	Transposase, IS4 family
11	87989	10730.3	gataattcaatttttattgatggt	AATAATTCAATTTTTATTGATGGt	Asp132Asn		2040	Transposase, IS4 family
11	88044	8770.44	ttctat	ttCCat	Tyr121His		2040	Transposase, IS4 family
11	88121	13823.8	ctt	cCt	Leu95Pro		2040	Transposase, IS4 family
11	88131	5333.83	gcccaagat	gcTCAAAat	AlaGlnAsp90AlaGlnAsn		2040	Transposase, IS4 family

17C9

Contig	Pos	Score	Ref_nt	Var_nt	Ref_aa_pos_change	Frameshift	Gene N°	Function
1	403394	3760.77	acgaaaaaatct	aCGAAAAAAATct	Thr120_Lys121fs	yes	385	Transposase, IS4 family
1	403435	12047.3	gcc	gTc	Ala109Val		385	Transposase, IS4 family
1	403568	2130.39	agt	Ggt	Ser65Gly		385	Transposase, IS4 family
3	125623	5724.65	gtc	gGc	Val41Gly		993	Transposase, IS4 family
7	80346	6415.04	tggatggtttac	tgGATGGATGGTttac	Trp2_Met3fs	yes	1570	Integral membrane protein
11	87939	10448.0	aagaaagta	AAGAAAAGta	Lys154_Lys155fs	yes	2040	Transposase, IS4 family
11	87948	13874.4	ttggtgcgg	ttTGTGTgg	LeuValArg151PheValTrp		2040	Transposase, IS4 family
11	87971	13993.9	agt	aAt	Ser145Asn		2040	Transposase, IS4 family
11	87989	8322.17	gataattcaatttttattgatggt	AATAATTCAATTTTTATTGATGGt	Asp132Asn		2040	Transposase, IS4 family
11	88044	6644.2	ttctat	ttCCat	Tyr121His		2040	Transposase, IS4 family
11	88121	9061.21	ctt	cCt	Leu95Pro		2040	Transposase, IS4 family
11	88131	3926.79	gcccaagat	gcTCAAAat	AlaGlnAsp90AlaGlnAsn		2040	Transposase, IS4 family
19	19253	6771.94						

20C9

Contig	Pos	Score	Ref_nt	Var_nt	Ref_aa_pos_change	Frameshift	Gene N°	Function
1	403394	1860.73	acgaaaaaatct	aCGAAAAAAATct	Thr120_Lys121fs	yes	385	Transposase, IS4 family
1	403435	11816.3	gcc	gTc	Ala109Val		385	Transposase, IS4 family
3	125623	3879.23	gtc	gGc	Val41Gly		993	Transposase, IS4 family
7	117763	15330.6						Intergenic region
7	80346	11520.1	tggatggtttac	tgGATGGATGGTttac	Trp2_Met3fs	yes	1570	Integral membrane protein
11	87939	13467.5	aagaaagta	AAGAAAAGta	Lys154_Lys155fs	yes	2040	Transposase, IS4 family
11	87948	18246.9	ttggtgcgg	ttTGTGTgg	LeuValArg151PheValTrp		2040	Transposase, IS4 family
11	87971	18140.6	agt	aAt	Ser145Asn		2040	Transposase, IS4 family
11	87989	11378.9	gataattcaatttttattgatggt	AATAATTCAATTTTTATTGATGGt	Asp132Asn		2040	Transposase, IS4 family
11	88044	10192.7	ttctat	ttCCat	Tyr121His		2040	Transposase, IS4 family
11	88121	14120.2	ctt	cCt	Leu95Pro		2040	Transposase, IS4 family
11	88131	5219.55	gcccaagat	gcTCAAAat	AlaGlnAsp90AlaGlnAsn		2040	Transposase, IS4 family

20C14

Contig	Pos	Score	Ref_nt	Var_nt	Ref_aa_pos_change	Frameshift	Gene N°	Function
1	403394	2411.24	acgaaaaaatct	aCGAAAAAAATct	Thr120_Lys121fs	yes	385	Transposase, IS4 family
1	403435	12433.2	gcc	gTc	Ala109Val		385	Transposase, IS4 family
1	403568	1058.79	agt	Ggt	Ser65Gly		385	Transposase, IS4 family
3	125623	7132.35	gtc	gGc	Val41Gly		993	Transposase, IS4 family
7	117763	12022.0						Intergenic region
7	80346	9216.03	tggatggtttac	tgGATGGATGGTttac	Trp2_Met3fs	yes	1570	Integral membrane protein
11	87939	11361.2	aagaaagta	AAGAAAAGta	Lys154_Lys155fs	yes	2040	Transposase, IS4 family
11	87948	15413.4	ttggtgcgg	ttTGTGTgg	LeuValArg151PheValTrp		2040	Transposase, IS4 family
11	87971	15071.6	agt	aAt	Ser145Asn		2040	Transposase, IS4 family
11	87989	8579.19	gataattcaatttttattgatggt	AATAATTCAATTTTTATTGATGGt	Asp132Asn		2040	Transposase, IS4 family
11	88044	7288.67	ttctat	ttCCat	Tyr121His		2040	Transposase, IS4 family
11	88121	12304.6	ctt	cCt	Leu95Pro		2040	Transposase, IS4 family
11	88131	5222.95	gcccaagat	gcTCAAAat	AlaGlnAsp90AlaGlnAsn		2040	Transposase, IS4 family
19	19253	9524.32						

B. SRS clones

17C1

Contig	Pos	Score	Ref_nt	Var_nt	Ref_aa_pos_change	Frameshift	Gene N°	Function
1	403394	3676.85	acgaaaaaatct	aCGAAAAAAATct	Thr120_Lys121fs	yes	385	Transposase, IS4 family
1	403435	11831.1	gcc	gTc	Ala109Val		385	Transposase, IS4 family
1	403568	2841.6	agt	Ggt	Ser65Gly		385	Transposase, IS4 family
3	125623	3995.43	gtc	gGc	Val41Gly		993	Transposase, IS4 family
7	80346	8331.0	tggatggtttac	tgGATGGATGGTttac	Trp2_Met3fs	yes	1570	Integral membrane protein
11	87939	13298.2	aagaaagta	AAGAAAAGta	Lys154_Lys155fs	yes	2040	Transposase, IS4 family
11	87948	15855.5	ttggtgcgg	ttTGTGTgg	LeuValArg151PheValTrp		2040	Transposase, IS4 family
11	87971	16275.6	agt	aAt	Ser145Asn		2040	Transposase, IS4 family
11	87989	7835.06	gataattcaatttttattgatggt	AATAATTCAATTTTTATTGATGGt	Asp132Asn		2040	Transposase, IS4 family
11	88044	6868.91	ttctat	ttCCat	Tyr121His		2040	Transposase, IS4 family
11	88121	9725.22	ctt	cCt	Leu95Pro		2040	Transposase, IS4 family
11	88131	2710.04	gcccaagat	gcTCAAAat	AlaGlnAsp90AlaGlnAsn		2040	Transposase, IS4 family
19	19253	7940.52						

17C2

Contig	Pos	Score	Ref_nt	Var_nt	Ref_aa_pos_change	Frameshift	Gene N°	Function
1	403394	3194.07	acgaaaaaatct	aCGAAAAAAATct	Thr120_Lys121fs	yes	385	Transposase, IS4 family
1	403435	11087.8	gcc	gTc	Ala109Val		385	Transposase, IS4 family
1	403568	200.647	agt	Ggt	Ser65Gly		385	Transposase, IS4 family
3	125623	7136.95	gtc	gGc	Val41Gly		993	Transposase, IS4 family
7	80346	8935.01	tggatggtttac	tgGATGGATGGTttac	Trp2_Met3fs	yes	1570	Integral membrane protein
11	87939	16297.9	aagaaagta	AAGAAAAGta	Lys154_Lys155fs	yes	2040	Transposase, IS4 family
11	87948	20364.8	ttggtgcgg	ttTGTGTgg	LeuValArg151PheValTrp		2040	Transposase, IS4 family
11	87971	21011.0	agt	aAt	Ser145Asn		2040	Transposase, IS4 family
11	87989	12312.4	gataattcaatttttattgatggt	AATAATTCAATTTTTATTGATGGt	Asp132Asn		2040	Transposase, IS4 family
11	88044	10146.4	ttctat	ttCCat	Tyr121His		2040	Transposase, IS4 family
11	88121	13868.4	ctt	cCt	Leu95Pro		2040	Transposase, IS4 family
11	88131	5855.47	gcccaagat	gcTCAAAat	AlaGlnAsp90AlaGlnAsn		2040	Transposase, IS4 family
19	19253	17467.3						

20C2

Contig	Pos	Score	Ref_nt	Var_nt	Ref_aa_pos_change	Frameshift	Gene N°	Function
1	403394	556.273	acgaaaaaatct	aCGAAAAAAATct	Thr120_Lys121fs	yes	385	Transposase, IS4 family
1	403435	9567.84	gcc	gTc	Ala109Val		385	Transposase, IS4 family
3	125623	1479.87	gtc	gGc	Val41Gly		993	Transposase, IS4 family
7	117763	14661.7						Intergenic region
7	80346	11587.3	tggatggtttac	tgGATGGATGGTttac	Trp2_Met3fs	yes	1570	Integral membrane protein
11	87939	11979.2	aagaaagta	AAGAAAAGta	Lys154_Lys155fs	yes	2040	Transposase, IS4 family
11	87948	15178.1	ttggtgcgg	ttTGTGTgg	LeuValArg151PheValTrp		2040	Transposase, IS4 family
11	87971	14636.2	agt	aAt	Ser145Asn		2040	Transposase, IS4 family
11	87989	7935.56	gataattcaatttttattgatggt	AATAATTCAATTTTTATTGATGGt	Asp132Asn		2040	Transposase, IS4 family
11	88044	5672.38	ttctat	ttCCat	Tyr121His		2040	Transposase, IS4 family
11	88121	14276.1	ctt	cCt	Leu95Pro		2040	Transposase, IS4 family
11	88131	5426.53	gcccaagat	gcTCAAAat	AlaGlnAsp90AlaGlnAsn		2040	Transposase, IS4 family
19	19253	14891.4						

20C6

Contig	Pos	Score	Ref_nt	Var_nt	Ref_aa_pos_change	Frameshift	Gene N°	Function
1	403394	2432.65	acgaaaaaatct	aCGAAAAAAATct	Thr120_Lys121fs	yes	385	Transposase, IS4 family
1	403435	12257.4	gcc	gTc	Ala109Val		385	Transposase, IS4 family
1	403568	1615.17	agt	Ggt	Ser65Gly		385	Transposase, IS4 family
3	125623	5223.67	gtc	gGc	Val41Gly		993	Transposase, IS4 family
7	117763	12361.2						Intergenic region
7	80346	11169.3	tggatggtttac	tgGATGGATGGTttac	Trp2_Met3fs	yes	1570	Integral membrane protein
11	87939	16271.8	aagaaagta	AAGAAAAGta	Lys154_Lys155fs	yes	2040	Transposase, IS4 family
11	87948	20177.4	ttggtgcgg	ttTGTGTgg	LeuValArg151PheValTrp		2040	Transposase, IS4 family
11	87971	20126.1	agt	aAt	Ser145Asn		2040	Transposase, IS4 family
11	87989	10902.3	gataattcaatttttattgatggt	AATAATTCAATTTTTATTGATGGt	Asp132Asn		2040	Transposase, IS4 family
11	88044	9020.43	ttctat	ttCCat	Tyr121His		2040	Transposase, IS4 family
11	88121	15486.6	ctt	cCt	Leu95Pro		2040	Transposase, IS4 family
11	88131	7121.32	gcccaagat	gcTCAAAat	AlaGlnAsp90AlaGlnAsn		2040	Transposase, IS4 family
19	19253	13848.4						

20C11

Contig	Pos	Score	Ref_nt	Var_nt	Ref_aa_pos_change	Frameshift	Gene N°	Function
3	125686	5479.66	ggt	gAt	Gly20Asp		993	Transposase, IS4 family
3	125693	5171.23	ggt	Tgt	Gly18Cys		993	Transposase, IS4 family
7	117763	18778.5						Intergenic region
7	80346	14960.1	tggatggtttac	tgGATGGATGGTttac	Trp2_Met3fs	yes	1570	Integral membrane protein
11	87939	13210.9	aagaaagta	AAGAAAAGta	Lys154_Lys155fs	yes	2040	Transposase, IS4 family
11	87948	17637.4	ttggtgcgg	ttTGTGTgg	LeuValArg151PheValTrp		2040	Transposase, IS4 family
11	87971	16740.1	agt	aAt	Ser145Asn		2040	Transposase, IS4 family
11	87989	7214.32	gataattcaatttttattgatggt	AATAATTCAATTTTTATTGATGGt	Asp132Asn		2040	Transposase, IS4 family
11	88044	5634.64	ttctat	ttCCat	Tyr121His		2040	Transposase, IS4 family
11	88121	16259.2	ctt	cCt	Leu95Pro		2040	Transposase, IS4 family
11	88131	5685.2	gcccaagat	gcTCAAAat	AlaGlnAsp90AlaGlnAsn		2040	Transposase, IS4 family
19	19253	22847.9						

20C21

Contig	Pos	Score	Ref_nt	Var_nt	Ref_aa_pos_change	Frameshift	Gene N°	Function
1	403394	2664.46	acgaaaaaatct	aCGAAAAAAATct	Thr120_Lys121fs	yes	385	Transposase, IS4 family
1	403435	11998.7	gcc	gTc	Ala109Val		385	Transposase, IS4 family
1	403568	1180.54	agt	Ggt	Ser65Gly		385	Transposase, IS4 family
3	125623	5644.9	gtc	gGc	Val41Gly		993	Transposase, IS4 family
7	117763	8506.89						Intergenic region
7	80346	7460.69	tggatggtttac	tgGATGGATGGTttac	Trp2_Met3fs	yes	1570	Integral membrane protein
11	87939	11594.4	aagaaagta	AAGAAAAGta	Lys154_Lys155fs	yes	2040	Transposase, IS4 family
11	87948	14585.7	ttggtgcgg	ttTGTGTgg	LeuValArg151PheValTrp		2040	Transposase, IS4 family
11	87971	14602.6	agt	aAt	Ser145Asn		2040	Transposase, IS4 family
11	87989	9391.69	gataattcaatttttattgatggt	AATAATTCAATTTTTATTGATGGt	Asp132Asn		2040	Transposase, IS4 family
11	88044	8126.29	ttctat	ttCCat	Tyr121His		2040	Transposase, IS4 family
11	88121	11770.1	ctt	cCt	Leu95Pro		2040	Transposase, IS4 family
11	88131	4639.58	gcccaagat	gcTCAAAat	AlaGlnAsp90AlaGlnAsn		2040	Transposase, IS4 family
19	19253	7977.6						

All intergenic mutations, non-synonymous mutations and insertions identified in the genomes of **A.** SSS and **B.** SRS clones are listed

The genome of the SSS clone 17C4 was used as reference in the variation analyses performed with PATRIC.

S, Susceptible; R, Resistant; SSS, Susceptible to phage vB_SauH_2002, phage 66, and the phage cocktail; SRS, Susceptible to phage vB_SauH_2002 and the phage cocktail and Resistant to phage 66.

The mutation only found in one SRS clone (20C11) but not in SSS clones is highlighted in red.

Supplemental Table S3. Summary of unique mutations identified in the genomes of **A.** SSS and **B.** SRS clones recovered from the vegetations of rats treated for 48 h with the phage cocktail/vancomycin combination.

A. SSS clones

Contig	Pos	Ref_nt	Var_nt	Ref_aa_pos_change	Frameshift	Gene N°	Function
1	403394	acgaaaaaatct	aCGAAAAAAATct	Thr120_Lys121fs	yes	385	Transposase, IS4 family
1	403435	gcc	gTc	Ala109Val		385	Transposase, IS4 family
1	403568	agt	Ggt	Ser65Gly		385	Transposase, IS4 family
3	125623	gtc	gGc	Val41Gly		993	Transposase, IS4 family
3	125686	ggt	gAt	Gly20Asp		993	Transposase, IS4 family
7	80346	tggatggtttac	tgGATGGATGGTttac	Trp2_Met3fs	yes	1570	Integral membrane protein
7	117763						Intergenic region
11	87939	aagaaagta	AAGAAAAGta	Lys154_Lys155fs	yes	2040	Transposase, IS4 family
11	87948	ttggtgcgg	ttTGTGTgg	LeuValArg151PheValTrp		2040	Transposase, IS4 family
11	87971	agt	aAt	Ser145Asn		2040	Transposase, IS4 family
11	87989	gataattcaatttttattgatggt	AATAATTCAATTTTTATTGATGGt	Asp132Asn		2040	Transposase, IS4 family
11	88044	ttctat	ttCCat	Tyr121His		2040	Transposase, IS4 family
11	88121	ctt	cCt	Leu95Pro		2040	Transposase, IS4 family
11	88131	gcccaagat	gcTCAAAat	AlaGlnAsp90AlaGlnAsn		2040	Transposase, IS4 family
19	19253						

B. SRS clones

Contig	Pos	Ref_nt	Var_nt	Ref_aa_pos_change	Frameshift	Gene N°	Function
1	403394	acgaaaaaatct	aCGAAAAAAATct	Thr120_Lys121fs	yes	385	Transposase, IS4 family
1	403435	gcc	gTc	Ala109Val		385	Transposase, IS4 family
1	403568	agt	Ggt	Ser65Gly		385	Transposase, IS4 family
3	125623	gtc	gGc	Val41Gly		993	Transposase, IS4 family
3	125686	ggt	gAt	Gly20Asp		993	Transposase, IS4 family
3	125693	ggt	Tgt	Gly18Cys		993	Transposase, IS4 family
7	80346	tggatggtttac	tgGATGGATGGTttac	Trp2_Met3fs	yes	1570	Integral membrane protein
7	117763						Intergenic region
11	87939	aagaaagta	AAGAAAAGta	Lys154_Lys155fs	yes	2040	Transposase, IS4 family

11	87948	ttggtgcgg	ttTGTGTgg	LeuValArg151PheValTrp	2040	Transposase, IS4 family
11	87971	agt	aAt	Ser145Asn	2040	Transposase, IS4 family
11	87989	gataattcaatttttattgatggt	AATAATTCAATTTTTATTGATGGt	Asp132Asn	2040	Transposase, IS4 family
11	88044	ttctat	ttCCat	Tyr121His	2040	Transposase, IS4 family
11	88121	ctt	cCt	Leu95Pro	2040	Transposase, IS4 family
11	88131	gcccaagat	gcTCAAAat	AlaGlnAsp90AlaGlnAsn	2040	Transposase, IS4 family
19	19253					

All intergenic mutations, non-synonymous mutations, and insertions identified in the genomes of **A.** SSS, and **B.** SRS clones are listed.

The genome of the clone 17C4-SSS was used as reference genome in the variation analyses performed with PATRIC.

S, Susceptible; R, Resistant; SSS, Susceptible to phage vB_SauH_2002, phage 66, and the phage cocktail; SRS, Susceptible to phage vB_SauH_2002 and the phage cocktail and Resistant to phage 66.

The mutation only found in SRS clones but not in SSS clones is highlighted in red.

SUPPLEMENTAL FIGURES

Supplemental Figure S1. Study design with sampling time points.

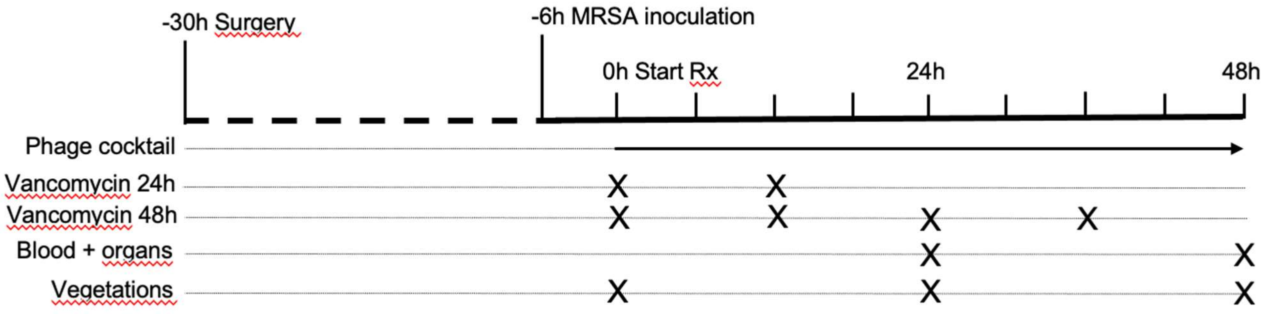

Supplemental Figure S2. Images of the patterns observed in diluted drop tests for *S. aureus* AW7 SSS and SRS clones isolated from the cardiac vegetations of rats treated with the phage cocktail/vancomycin combination for 48 h and from which the genome was fully sequenced. **A.** AW7 and SSS clones, and **B.** SRS clones. On each Petri dish, the phages vB_SauH_2002, the phage 66, and the phage cocktail were deposited from left to right, respectively. S, susceptible; R, resistant.

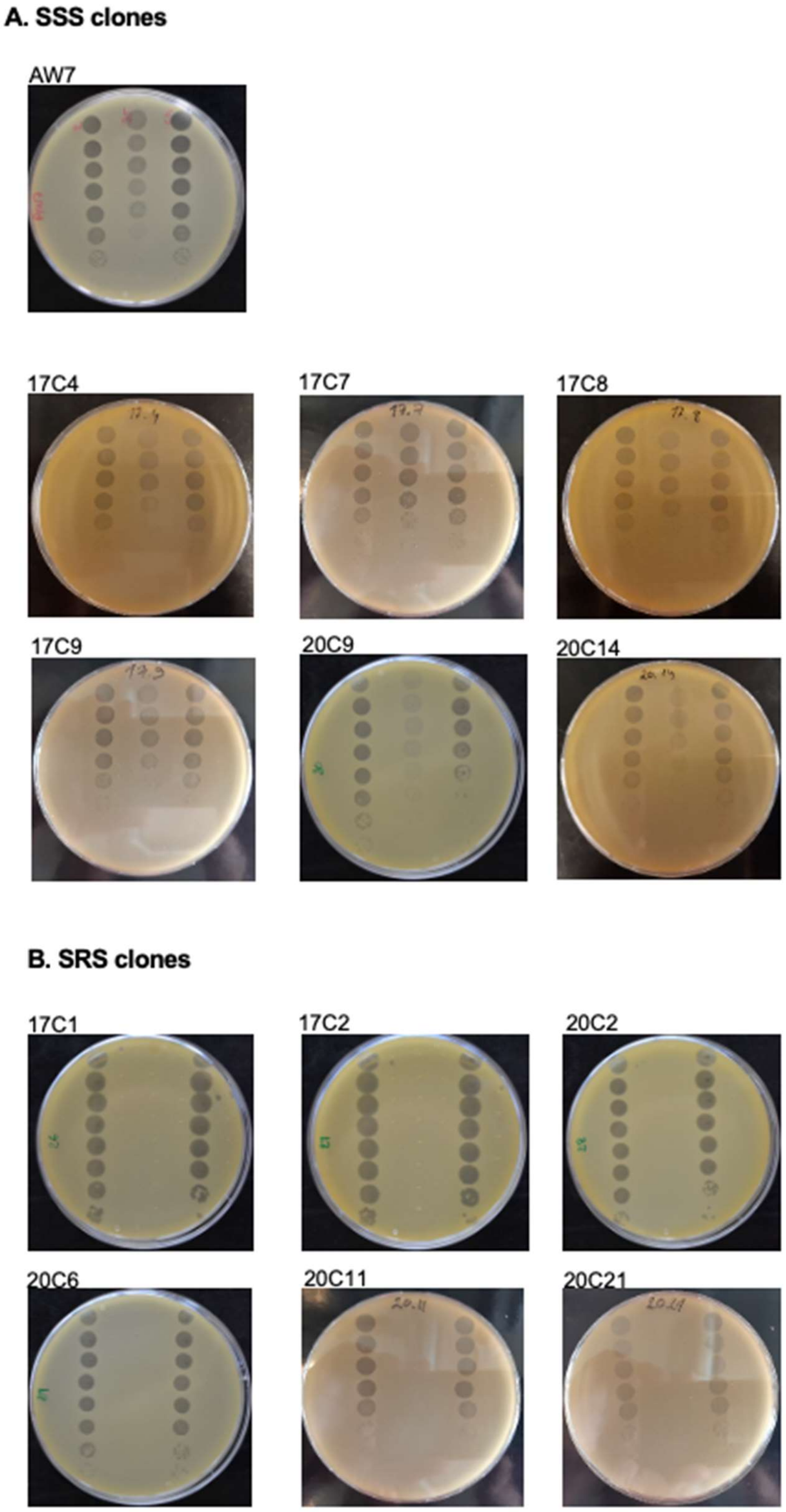

Supplemental Figure S3. Activity of **A.** phage vB_SauH_2002 and **B.** phage 66 against planktonic *S. aureus* AW7. Turbidity assays were performed in 96-microtiter plates with ca. 6 log_{10} CFU/mL of cells challenged with phages alone at different MOIs. Control without phages (closed black circles); phages at MOI = 0.01 (open circles), MOI = 0.1 (open diamonds), MOI = 1 (open triangles), MOI = 10 (closed squares), and MOI=100 (closed triangles). CFU, colony forming unit; MOI, multiplicity of infection; MIC, minimum inhibitory concentration.

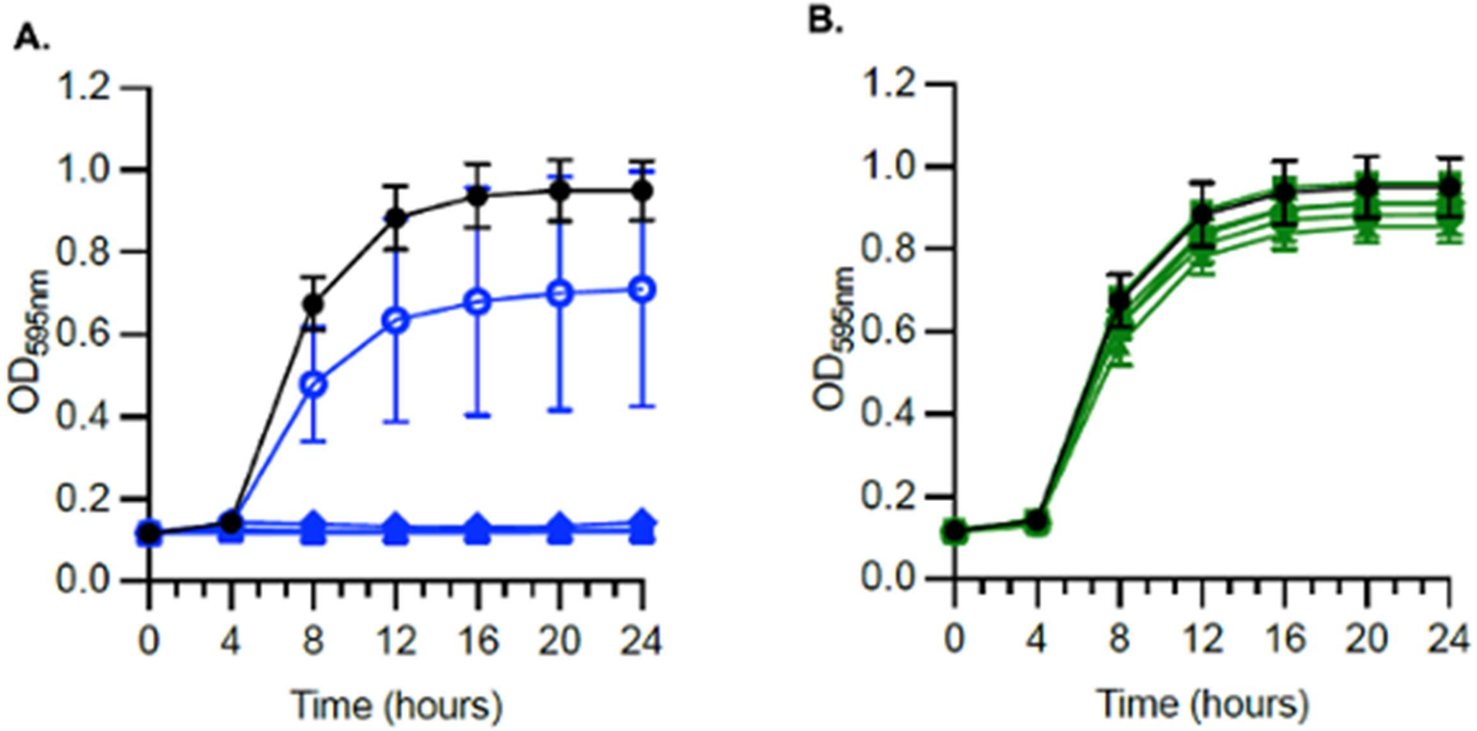

Supplement: Supplementary file 1 [file viruses-14-01792-s001.zip › viruses-1828778-SM.pdf]
